# Anterior subcutaneous internal fixation for treatment of unstable pelvic fractures

**DOI:** 10.1186/1756-0500-7-133

**Published:** 2014-03-08

**Authors:** Max J Scheyerer, Stefan M Zimmermann, Georg Osterhoff, Simon Tiziani, Hans-Peter Simmen, Guido A Wanner, Clèment ML Werner

**Affiliations:** 1 Department of Surgery, Division of Trauma Surgery, University Hospital, Zurich, Switzerland

**Keywords:** Pelvic ring fracture, Rupture of the symphysis pubis, Internal fixator, Anterior subcutaneous fixator

## Abstract

**Background:**

Fractures of the pelvic ring including disruption of the posterior elements in high-energy trauma have both high morbidity and mortality rates. For some injury pattern part of the initial resuscitation includes either external fixation or plate fixation to close the pelvic ring and decrease blood loss. In certain situations – especially when associated with abdominal trauma and the need to perform laparotomies – both techniques may put the patient at risk of either pintract or deep plate infections. We describe an operative approach to percutaneously close and stabilize the pelvic ring using spinal implants as an internal fixator and report the results in a small series of patients treated with this technique during the resuscitation phase.

**Findings:**

Four patients were treated by subcutaneous placement of an internal fixator. Screw fixation was carried out by minimally invasive placement of two supra-acetabular iliac screws. Afterwards, a subcutaneous transfixation rod was inserted and attached to the screws after reduction of the pelvic ring. All patients were allowed to fully weight-bear. No losses of reduction or deep infections occurred. Fracture healing was uneventful in all cases.

**Conclusion:**

Minimally invasive fixation is an alternative technique to stabilize the pelvic ring. The clinical results illustrate that this technique is able to achieve good results in terms of maintenance of reduction the pelvic ring. Also, abdominal surgeries no longer put the patient at risk of infected pins or plates.

## Findings

Pelvic injuries represent a relatively small rare injury, making up between 2–8 per cent of all fractures of the human body [1,2]. They usually result from massive-, and commonly life-threatening external forces as can be encountered following motorbike accidents or falls from a great height. This sort of high-energy trauma resulting in predominantly unstable pelvic injuries is commonly associated with a young patient collective between the age of 15 and 30 years. Stable pelvic fractures are usually attributed to the elderly with an age range around seventy [3]. Depending on the direction of the acting force, either predominantly ligamentous or osseous lesions result, which affect the integrity of the pelvic ring.

The low incidence of these injuries is in great contrast to the high mortality rate of 5-20%. Some studies even report lethality as high as 60% [4-8]. The reason for this is attributed to the high level of blood loss, which leads to hemodynamic shock and multi- organ failure [8]. Beside the fracture plain itself, the main source of bleeding is also due to lesions of the pelvic venous plexus. In up to 25% per cent of the cases, arterial haemorrhaging can be noted, especially from branches of the A. iliaca interna [9,10]. An injury to the pelvic ring not only leads to a loss of stability, but also increases the volume of the intra-pelvic compartment. This impedes spontaneous tamponade of bleeding. Frequent associated ruptures of the musculature of the pelvic floor may further contribute to this [11]. Beside general measures of circulatory stabilisation, reconstruction of pelvic anatomy and thereby restoration of a stable, non-expandable compartment is a main goal in patient management. Especially the reconstruction of the posterior pelvic ring is hereby of great importance. On the other hand, biomechanical studies have also shown that additional osteosynthesis of anterior lesions significantly improves stability [12,13]. It seems that the structures of the abdominal wall inserting at the anterior structures of the pelvic ring mainly account for this [14]. For this reason it has been proven necessary to re-establish the integrity of the anterior pelvic ring as well especially in case of an unstable type C lesion.

In an emergency setting, several primary measures can be taken to address this issue such as the application of a pelvic harness, a pelvic c-clamp or external fixateur. In case of persistent haemodynamically relevant bleeding, additional tamponade of the lesser pelvis or angiologic selective embolisation may become necessary.

Associated lesions of the urogenital- and digestive system are frequent in severely injured patients. They most often necessitate prompt surgical treatment as well, which is usually performed through a median laparotomy [3]. When treating polytraumatized patients with abdominal lesions, repeated surgical interventions are frequently necessary. In such cases, an external fixateur not only directly interferes with the operative field, but has also been shown to increase the risk of pin tract infections by up to 50% [15].

Minimally invasive surgical techniques may furthermore be of great advantage concerning complication rates in cases of large pelvic defects, multiple- or comminuted fractures of the anterior pelvic ring, coagulopathies and history of previous hip or abdominal surgery [16].

A novel solution shall be discussed in the following as an alternative to current treatment modalities such as plating of the symphysis or use of an external fixateur. Implants previously established in spine surgery are hereby used to construct a fixateur interne [16-18]. The technique has previously been described and has had a multicentre trial already [19]. However, we used the technique with several modifications. Advantage of this technique is, that contrary to the external fixateur, the screws as well as the connecting rod are placed subcutaneously in a separate compartment and thus without any contact to a possible laparotomy site and with no extracorporal components.

### Methods

The aim of the operation is to restore definitive ventral stability by applying an internal fixateur. The target population are patients suffering from pelvic ring injuries with transsymphysial instability with an additional necessity for laparotomy or contraindications for ventral symphysial plating. Due to the minimally-invasive approach, less soft tissue damage is caused compared to conventional open reduction and plate fixation. The subcutaneous positioning of the implant is isolated from an intra-abdominal operation site; thereby the risk of bacterial colonisation and consecutive soft tissue infection is minimized. Additionally, the stability of the anterior pelvic ring is increased through a shortened lever arm compared to an external fixateur. The advantages and disadvantages are summarised in Table 1.

**Table 1:** Advantages and disadvantages

**Advantages**	**Disadvantages**
Minimally-invasive approach with minimal soft tissue damage	Danger of injury to the A./V. epigastrica superficialis and A./V. circumflexa ilium superficialis
No extensive soft tissue dissection necessary	Danger of perforation of the abdominal muscles and urinary bladder
Short operation time (average total duration of procedure 30-45 min)	Decreased stability compared to open reduction and plate fixation in pure open-book injuries
Immediate postoperative mobilisation	Possibly interposed soft tissue within the fracture site cannot be removed
No contact with other surgical wounds	Fracture fragments causing neural or organ compression cannot be removed
No open pin tracts compared to the external fixateur	Implant removal always in operative theatre (compared to external fixateur)
Minimal blood loss	
No restrictions regarding patient anatomy; easy use even in morbidly obese patients	
No communication of implants with intra-abdominal compartment	
Biomechanically proven superior stability compared to external fixateur [20]	
Further diagnostics such as computed tomography are not interfered with	
Reversible procedure: Salvage procedures are still available	

### Indications

Indications for this procedure are well described in literature [16-19] and summarized below. Stabilisation of transpubic instability [21]. Stabilisation of transsymphysial instability with gaping symphysis (>2 cm) [21]. As additional procedure in combination with primary dorsal stabilisation in cases of complex pelvic ring fractures [17,21]. Temporary volume reduction within the lesser pelvis in cases of complex pelvic ring fractures especially when combined with concomitant abdominal lesions necessitating operative treatment. In cases of extensive soft tissue defects following crush injuries or open fractures. Severely injured patients with expected prolonged intensive care treatment in an effort to reduce risk of infection compared to external stabilisation devices and facilitate every-day care and mobilization. Use in cases where in a later phase prone patient positioning is necessary such operative treatment of spine fractures. Use in cases of Coagulopathy due to reduced blood loss compared to open procedures.

### Contraindications

Acetabular fractures with involvement of the anterior column. Stable pelvic ring fractures. Pregnancy.

### Patient education

Patient education and obtaining of informed consent is frequently not possible due to concomitant injuries. Otherwise the complications listed in Table 2 should be mentioned.

**Table 2:** General and specific complications of the technique

**General complications**	**Specific complications**
Infection	Injury of A./V. femoralis as well as epifascial blood vessels when introducing the transfixation rod
Wound healing disturbances	Nerve injuries, specificall N. cutaneus femoris lateralis and N.femoralis [22]
Thrombosis/embolism	Perforation of abdominal muscles with injury of surrounding structures and organs [22]
	Implant malpositioning/loosening of the supra-acetabular iliac screws
	Perforation of the hip joint
	Secondary operative intervention for later implant removal compared to one single planned intervention in primary plate fixation

### Preoperative considerations

**Figure 1:**
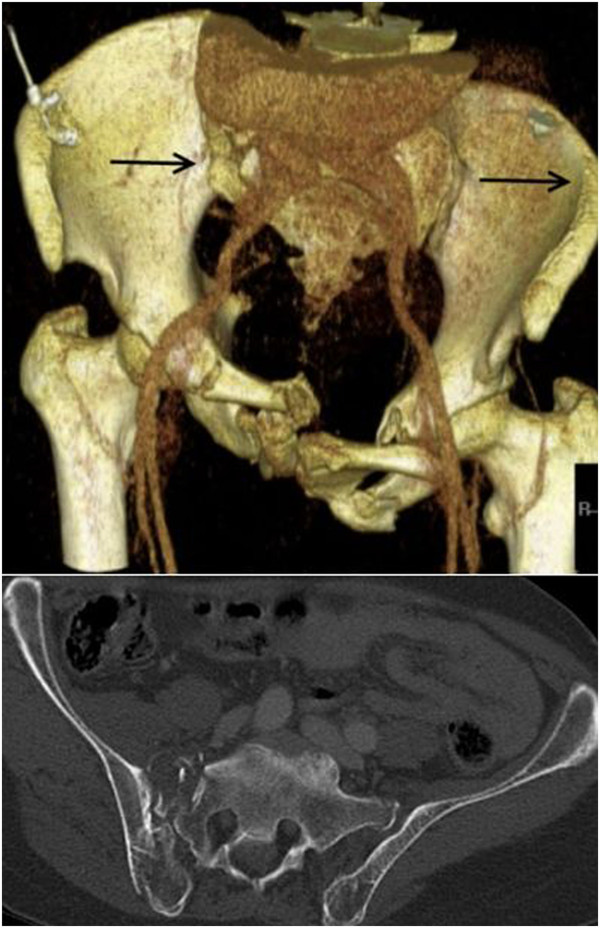
**A 65 year**-**old patient was hit by a car as pedestrian.** Computed tomography revealed a lateral compression type III pelvic ring fracture. The arrows mark the direction of force. In addition to restoration of pelvic ring instability, a median laparotomy was necessary duet to a concomitant laceration of the spleen Moore type II as well as an extra-peritoneal urinary bladder rupture.

Antero-posterior pelvic radiogram, oblique views (inlet- and outlet view), possibly computed tomography (Figure 1). Patient positioning allowing for later intra-operative fluoroscopic ap-, ala- and obturator views. Preoperative planning of expected rod length for internal fixation.

### Instruments and implants

Legacy Iliac Multiaxial (FA Medtronic, Inc, Tolochenaz, Switzerland) Quadrant wound retractor (FA Medtronic, Inc, Tolochenaz, Switzerland) Longitude instruments (FA Medtronic, Inc, Tolochenaz, Switzerland).

### Anaesthesia and patient positioning

General anaesthesia. Supine patient position. Adequate and generous draping ensuring with specific care to the genital area.

### Operation technique

**Figure 2:**
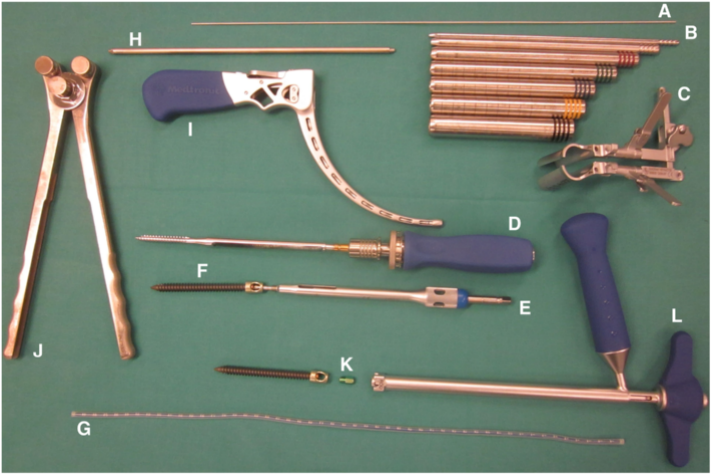
A compilation of necessary instruments and implants.

The following instruments are required on a separate side table: a) A long, 6.3 mm thick K-wire, b) dilatation trocars, c) quadrant wound retractor, d) thread cutting tool, e) screw driver, f) two iliac screws 6.5 mm, g) flexible test rod, h) longitude rod, i) mounted instrumentarium for insertion and manipulation of longitude rod, j) rod bending devices, k) covering caps mounted on the corresponding screw driver, l) counterholder (Figure 2).

**Figure 3:**
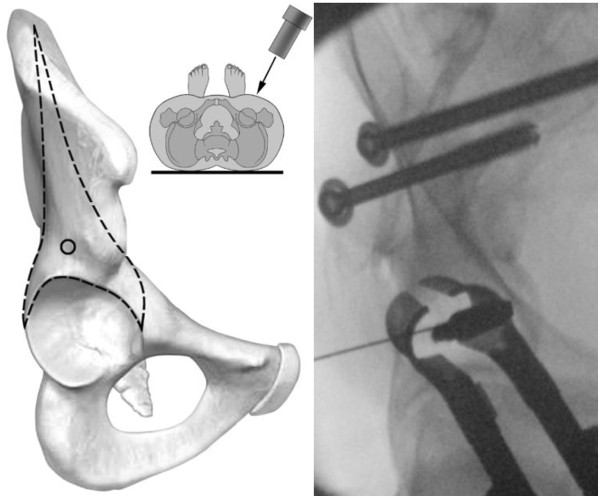
Intraoperative vision of the entry points for the iliac screws.

**Figure 4:**
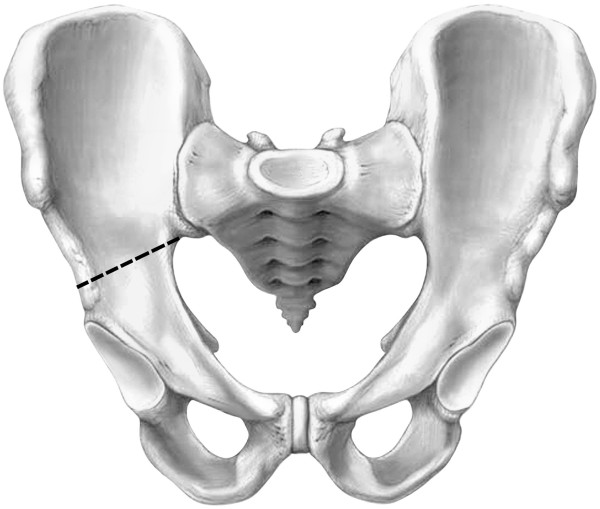
**Positioning of K**-**wires in 20 degrees cranio**-**caudal and 30 degrees medial.**

With use of fluoroscopy, the first step is to mark the entry points for the iliac screws, which are located in the centre of the supra-acetabular triangle. For this, the fluoroscope needs to be directed roughly 30° caudally and in 30° inclination in latero-medial direction (Figure 3) in order to obtain an outlet- and obturator view. Skin incisions are performed on both sides roughly 3 cm medial and 4 cm distal of the easily palpable spina iliaca anterior superior. Following the skin incisions, blunt dissection is performed up to the spina iliaca anterior inferior, which can be easily palpated with a blunt clamp. During dissection, extra care needs to be taken to not injure the N. cutaneus femoris lateralis. The thinnest trocar of the Quadrant system is then introduced as drill guide. A security margin of at least 1,5-2 cm to the radiological hip joint line is to be adhered to, since the insertion of the hip capsule is close by [23]. Following this, a 6,3 mm K-wire is introduced through the cannulated trocar. As a general guideline, drilling direction should be aimed 20 degrees cranio-caudal and 30 degrees medial (Figure 4). Supra-acetabular K-wire placement is ensured through fluoroscopic control as the wire is advanced toward the dome of the incisura ischiadica major.

**Figure 5:**
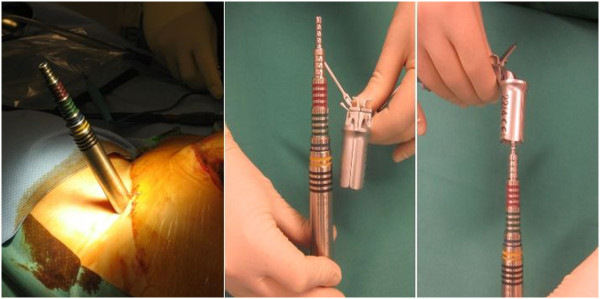
**Dilation**-**sleeves inserted over the K**-**wire to widen the access.**

**Figure 6:**
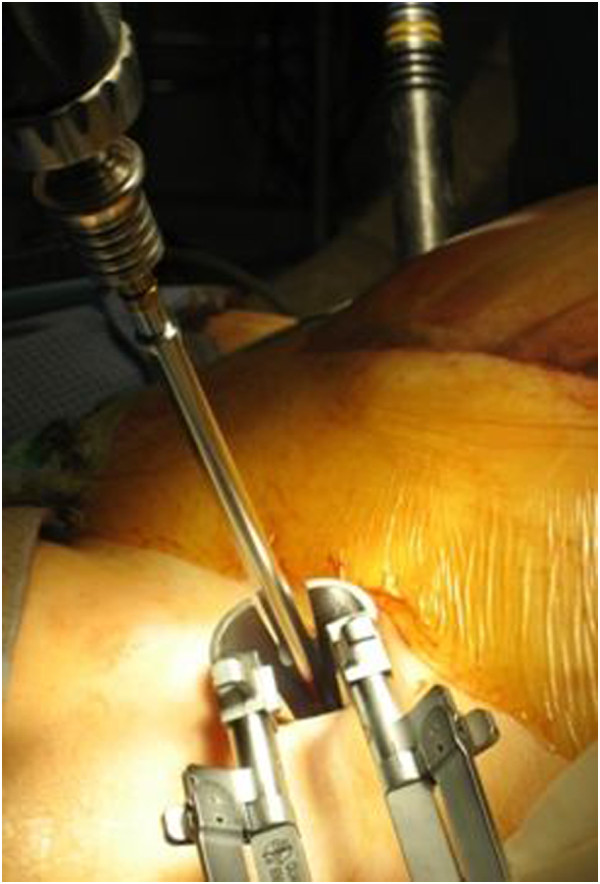
Insertion of a 6,5 mm thread cutting device.

**Figure 7:**
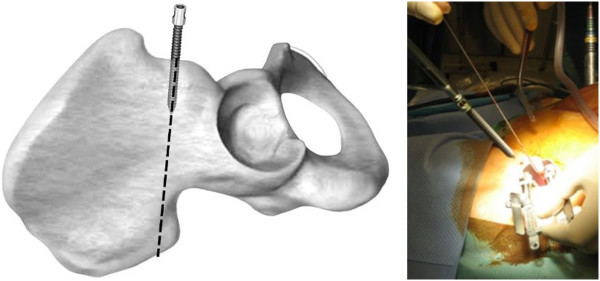
Placement of Legacy Iliac MAS screw under fluoroscopic guidance.

Dilation-sleeves inserted over the K-wire are used in an ascending order (largest = black sleeve) to widen the access (Figure 5). The quadrant wound retractor is now inserted over the dilatation sleeves. Using the K-wire as guidance, a thread cutting device is inserted (Figure 6). The Legacy Iliac MAS screw is now placed under fluoroscopic guidance (Figure 7).

**Figure 8:**
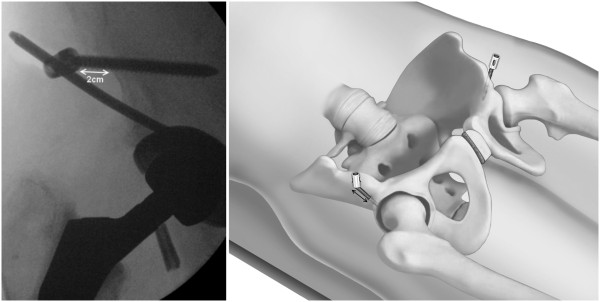
A distance of roughly 2 cm should be kept between the bone and screw head in order to avoid later vessel compression following introduction of the transfixation rod.

**Figure 9:**
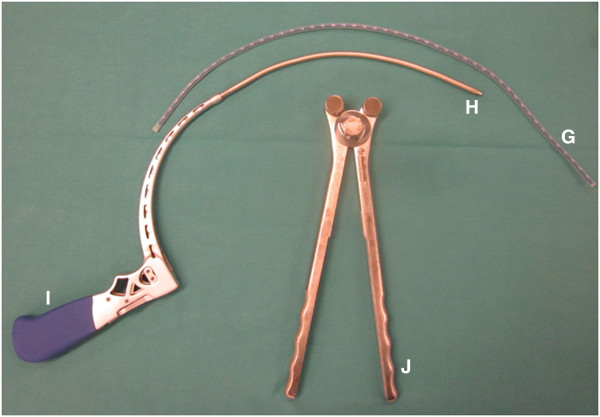
Bending instruments are used to mould the rod accordingly.

**Figure 10:**
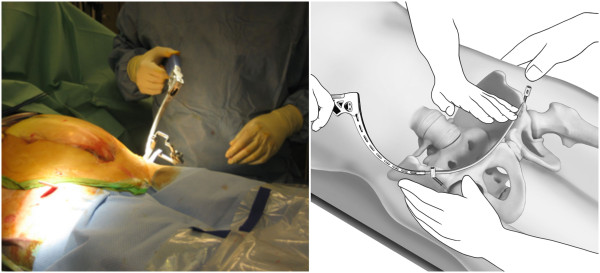
The rod is inserted through one of the supra-acetabular incisions and gently advanced in the subcutaneous layer.

It is important that a distance of roughly 2 cm is kept between the bone and screw head in order to avoid later vessel compression following introduction of the transfixation rod (Figure 8). The flexible test rod is used to define the desired length of the Longitude rod. For easier fixation, the rod is mounted to the Longitude rod holder before bending the rod. Bending instruments are used to mould the rod accordingly (Figure 9). The rod is inserted through one of the supra-acetabular incisions and gently advanced in the subcutaneous layer (Figure 10). The risk of rod malpositioning can be minimized as correct rod advancement is ensured by placing one hand on the patient’s abdominal wall for palpation. When the rod has reached the contralateral side, it can be grasped with the rod grasping forceps and is then guided through the hole in the iliac screw. Correct rod positioning is then verified and documented fluoroscopically and femoral as well as epifascial vessels are checked for any possible compression with a sterile Doppler ultrasound device [18,19].

**Figure 11:**
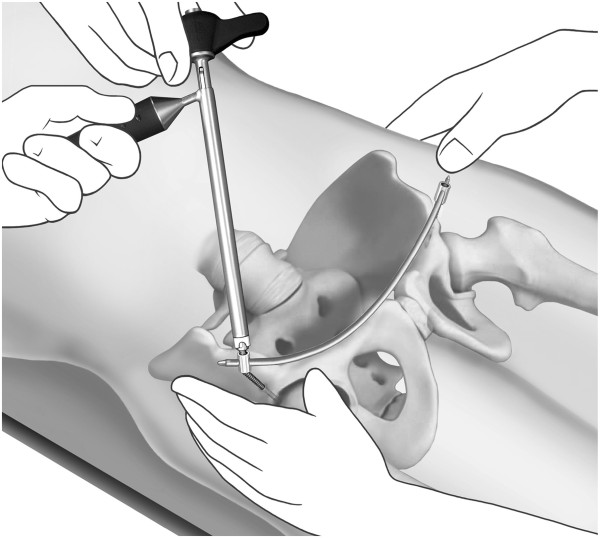
**After positioning of the rod**, **it is fixed to the iliac screw on one side.** Then the pelvic ring injury is reduced through lateral compression and if necessary leg traction and internal rotation.

When the desired position of the rod is achieved, it is fixed to the iliac screw on one side with the green sealing cap. Following this, the pelvic ring injury is reduced through lateral compression and if necessary leg traction and internal rotation (Figure 11). The assistant manually maintains reduction as the surgeon fixes the rod to the iliac screw again using the green sealing cap. With the counterholder, the top of the caps can now be broken off.

In the illustrated case below, the almost anatomically-, yet insufficiently reduced symphysis was accepted as a concomitant fracture of the right pubic ramus was present and anatomical positioning of the ilio-sacral joint had been achieved. As the patient remained asymptomatic, further open anterior revision was not necessary later on.

**Figure 12:**
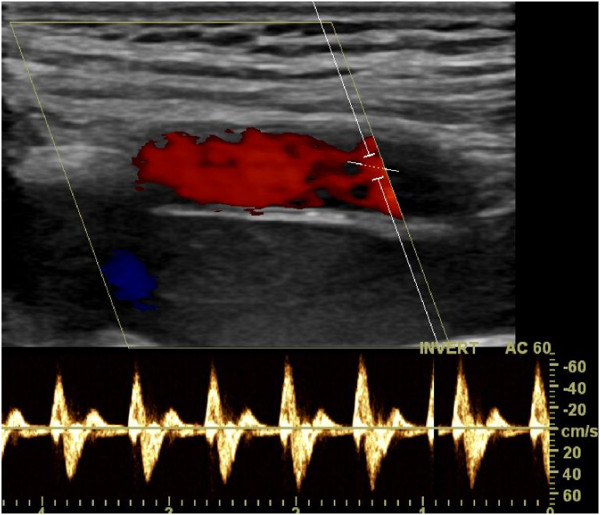
Postoperatively a Doppler ultrasound in neutral position as well as in 90 and 120 degrees of flexion is performed to rule out possible compression of femoral and epifascial vessels.

In order to rule out possible compression of femoral and epifascial vessels, a Doppler ultrasound is performed postoperatively in a routine manner with the hip in neutral position as well as in 90 and 120 degrees of flexion (Figure 12).

### Distinctive features of the operative procedure

Use of the well-established external fixateur with supra-acetabular pins and an extracorporal connecting rod system may provide good primary stability. It is however not sufficient for complete patient mobilisation. The internal fixation system described in this manuscript has specific biomechanical advantages. The lever arm on the supra-acetabular iliac screws is reduced substantially through the subcutaneous rod placement therefore decreasing the acting forces on the fixation points [17,24]. This leads to increased stiffness and might encourages bone healing. Furthermore we think that the built-in hole within the screw head, as can be seen on Figure 2, is another advantage of the present used iliac screws. It can be used as direct connection to the fixation rod, which facilitates assembly compared to the U-shaped heads of the pedicle screw. So far no problems could be observed to thread the rod.

As entry point for the screws, we choose the stable corridor within the supra-acetabular bone as described for pin placement of external fixation devices by Gänsslen et al. [12]. In contrast to the work of Kuttner et al., surrounding muscle insertions do not need to be mobilised when choosing this entry point [16]. However, great care must be taken to not injure the N. cutaneus femoris lateralis. A temporary nerve irritation was observed by Vaidja et al. in a large patient collective in up to 30 per cent of the included 91 patients [25]. However, symptoms resolved spontaneously over the course of time in all but one case. Through use of the dilatation sleeves described in our manuscript, blunt preparation of the entry points and use of the quadrant wound retractor is aimed at further reducing such complications. No such nerve irritations were observed in our patient collective.

### Implant removal

The internal fixateur serves as definitive stabilisation of the anterior pelvic ring and should be left in situ for at least three months. Following this time period, implant removal is effortless and achieved using the same incisions as with implantation. Compared to implant removal following symphysial plating, soft tissue damage is substantially reduced which leads to shorter hospitalisation times.

### Postoperative care

**Figure 13:**
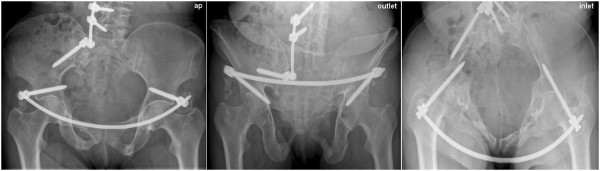
**Conventional x**-**ray images** (**anterior**-**posterior**, **inlet and outlet**) **one month postoperatively following iliolumbar transfixation and percutaneous ventral pelvic ring stabilisation in a 65 year**-**old patient with LC III type fractures (compare Figure**1**).** In the row below the follow-up images can be seen.

If necessary intensive care treatment is recommended. As soon as possible a sufficient thrombosis prophylaxis in this high-risk patients must be initiated. Further posterior pelvic ring lesions or other additional injuries should be treated, as soon as the patients’ condition permits. Beside regular wound checks the nursing is unrestricted with regard to patients positioning. Weight bearing of lower extremities depends to additional lesions of the pelvic ring. Full weight bearing is possible in cases of isolated anterior pelvic ring injuries. Postoperative radiological documentation with inlet and outlet views and further radiological documentation of osseous consolidation and maintenance of reduction over the course of time is necessary (Figure 13).

### Potential errors, dangers, complications

Injury of the hip joint due to unintended intra-articular placement of the iliac screws [22]. Perforation of the abdominal wall while subcutaneous advancement of the fixation rod is in progress with risk of organ lesion (iatrogenic urinary bladder-, sigma-, coecum lesion or injury to the small intestine). Risk of laceration of the A./V. femorales as well as epifascial vessels when advancing the fixation rod. Risk of secondary nerve damage due to compression following closed reduction in cases of simultaneous sacrum fractures. Irritation of the N. cutaneus femoris lateralis [25]. Loosening or dislocation of the fixation rod with loss of reduction [25]. Wound infection. Heterotopic ossification (Figure 13) [25].

### Short term results

Beside the presentation of the surgical procedure we evaluated the short-term results of the first four patients treated with this novel technique during the resuscitation phase. Abovementioned authors were the attending physicians for each of the patients. Due to the retrospective nature of the investigation and the current local regulations no further approval of the local ethics committee or the patients was necessary. All data were further used exclusively in an anonymized form.

## Results

### Case report

The following is an exemplary description of the case of a 65 year-old patient, who as a pedestrian was hit by a car from the side. Consent to publish details of the case were obtained from the patient before publication. Initial diagnostic work-up revealed blunt thoracic trauma, a pelvic ring fracture type LC III (Figure 1), a spleen laceration Moore type II as well as a rupture of the urinary bladder. The above mentioned intra-abdominal lesions required a median laparotomy for adequate treatment. Within the same session, the pelvic ring was stabilized with an internal fixateur. It was chosen over an external fixation device since multiple abdominal follow-up procedures were scheduled and an external device would have interfered with the surgical site. With the internal fixateur in place, all necessary abdominal procedures could be readily performed and postoperative intensive care was facilitated. Even prone patient positioning for treatment of the blunt thoracic trauma was possible.

Following intensive care treatment, the patient could be mobilized nearly pain-free by using two crutches. Transition to full weight bearing was initiated after six weeks. Postoperative radiological documentation demonstrated satisfactory results regarding reduction, which was confirmed in the consecutive follow-up examinations (Figure 13).

Asymptomatic heterotopic ossification occurred over the course of time around the iliac screws.

The implant was removed three months postoperatively in a 25 minute operation. In the following clinical follow-ups the patient remained free of pain.

Beside the aforementioned case a total of four patients suffering from unstable pelvic ring fractures were treated with the above described internal fixation within our patient collective between September 2011 and January 2012.

Two patients had suffered a lateral compression (LC) type III injury according to Young/Burgess, one pelvic ring lesion was classified as combined mechanism (CM) and one as anterior-posterior compression (APC) [26].

The mean patient age was 66 (range 55–84); three patients were male and one was female. All patients were poly-traumatized with an average ISS (Injury Severity Score) of 34 (17–48). The cause of injury was traffic related in all four cases, whereby three of the patients were hit by a car as pedestrians and one patient was injured during a motorcycle crash. An emergency-laparotomy upon admission was necessary due to concomitant intestinal or urogenital injuries in two of the four patients. Another patient underwent laparotomy later on.

The indication for stabilisation with an internal fixateur was pelvic ring instability. The procedure was performed on average 2,5 days after the accident (range 0–5 days) and operation time was 50 minutes (45–60 minutes). Two patients additionally underwent stabilisation of the posterior pelvic ring, one patient had succumbed to his serious injuries before reconstruction of the posterior pelvic ring was possible. There were no intraoperative complications. An unaltered flow of the A./V. femorales was documented with Doppler ultrasonography in all cases (Figure 12).

The postoperative phase was uneventful as well. There were no wound infections and all follow-up surgical procedures- even treatment of spine fractures with the patient in prone position- could be performed without limitations. Contrary to the work of Vaidja et al., who described nerve irritations in up to 30 per cent of the 91 included patients, no irritations of the N.cutaneus femoris lateralis were observed in our patient collective [25].

All patients were allowed partial weight bearing with 15 kg postoperatively due to concomitant sacrum fractures. The following transition to full weight bearing was conducted using two crutches and was uneventful. In one case, heterotopic ossification occurred around the iliac screw without causing any symptoms. One patient found the implant bothersome.

**Figure 14:**
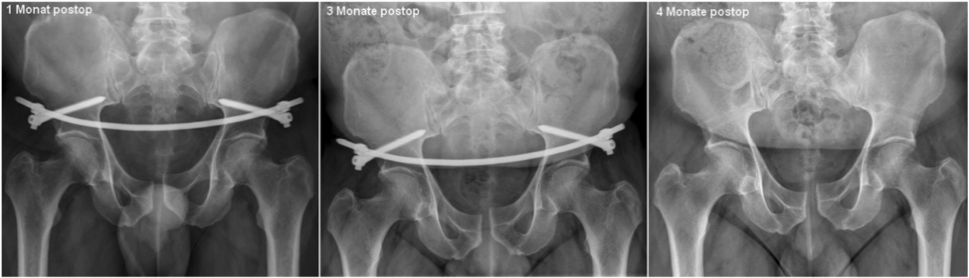
**X**-**ray images following treatment of an APC II injury in a 61 year**-**old patient which had been sustained in a motorcycle crash.** Due to an additional spleen laceration a laparotomy was necessary. The pelvic ring instability was treated with an internal fixateur.

No secondary loss of reduction was recorded in any of the follow-up consultations. A slight persistent diastasis of the symphysis was recorded in one of the patients (Figure 14). However, since the patient was free of any symptoms, revision surgery with open reduction did not become necessary.

The implants were removed in all patients after three to four months.

In conclusion, the internal fixateur represents a novel minimally-invasive procedure for definitive treatment of anterior pelvic ring instability. Depending on the scenario, it may have specific advantages compared to current treatment modalities. Noteworthy hereby are the preservation of soft tissue, increased stability and facilitated patient care due to the subcutaneous location of the implant.

Unlike the external fixateur or anterior plating, the risk of infection does not increase when the internal fixateur is applied in cases where a simultaneous laparotomy is necessary. However, due to the small case series of four isolated patients further investigations are needed to confirm the observed good clinical effectiveness.

## Competing interests

The authors declare that they have no competing interests.

## Authors’ contributions

MJS, Study conception and design; Acquisition of data; Analysis and interpretation of data; Drafting of manuscript; SMZ, Drafting of manuscript; GO, Acquisition of data; Analysis and interpretation of data ST, Acquisition of data; Analysis and interpretation of data GAW, Study conception and design; Critical revision HPS, Study conception and design; Critical revision CMLW, Study conception and design; Analysis and interpretation of data; Critical revision. All authors read and approved the final manuscript.
